# A Survey of Patient Demographics in Inflammatory Skin Disease Case Reports

**DOI:** 10.2196/49070

**Published:** 2023-09-25

**Authors:** Ross O'Hagan, Stella A Caldas, Patrick M Brunner, Benjamin Ungar

**Affiliations:** 1 Department of Dermatology Icahn School of Medicine at Mount Sinai New York, NY United States

**Keywords:** inflammatory skin diseases, acne, alopecia areata, atopic dermatitis, psoriasis, rosacea, demographic, survey, case reports, case report, skin, inflammatory, inflammation, dermatitis, dermatology, demographics

## Abstract

Case reports serve many functions in the medical literature. We explore patient demographics in case reports for common inflammatory skin diseases.

## Introduction

What functions do case reports serve in the medical literature and practice? Their functions are numerous, including the dissemination of unique clinical observations, novel treatment approaches, and hypothesis generation [1]. While individual case reports have inherent limitations such as publication bias and limited scope, previous work has shown situations in which meta-analyses of case reports and formal clinical studies agree [2]. By aggregating large numbers of published case reports, the impact of clinical and treatment outliers (ie, “black swan” events [3]) reported in the dermatology literature can potentially be dampened. However, little work has been carried out to assess which diseases and demographics have a greater number of published case reports associated with them. In this paper, we use a novel data set to explore the potential biases in case report publications in inflammatory skin conditions by disease for demographic factors.

## Methods

In this study, we evaluated the frequency of case reports of 5 common inflammatory skin diseases using PubMed Central Patients (PMC-Patients), a collection of 167,000 patient summaries extracted from case reports [4]. Reports on patients with diseases of interest were collected using string match for the disease, and demographic information of the patients in the identified case reports was extracted from PMC-Patients. All analyses and figures were generated using R (version 4.2.2; R Core Team). This study did not require institutional review board approval.

## Results

**Figure 1:**
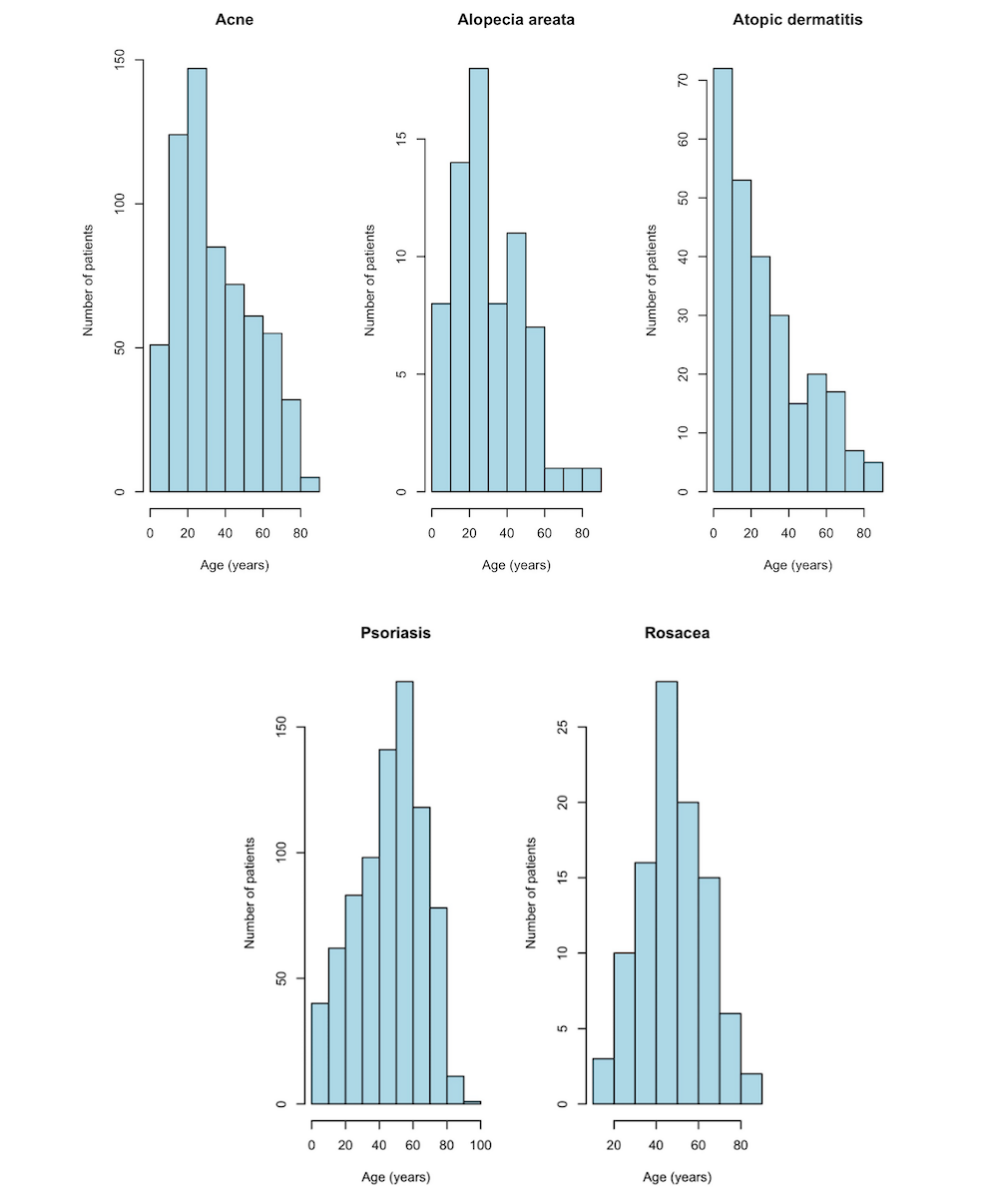
Histograms of patient age by inflammatory skin disease type in case report literature.

Case reports were found for patients with acne (n=632), alopecia areata (AA; n=69), atopic dermatitis (AD; n=259), psoriasis (n=800), and rosacea (n=100). We found that AD had the smallest percentage of female patients (n=107, 41.3%), and rosacea had the largest (n=58, 58%). Female patients accounted for 53.5% (n=338), 49.3% (n=34), and 43.5% (n=348) of case reports on acne, AA, and psoriasis, respectively. The mean age was 34.6 (SD 20.1) years for patients with acne, 30.9 (SD 18.1) years for patients with AA, 27 (SD 22.2) years for patients with AD, 46.4 (SD 19.8) years for patients with psoriasis, and 48.4 (SD 15) years for patients with rosacea. Plotting histograms of patient ages indicated that rosacea cases had a normal distribution, AD and acne had a right-sided skew, psoriasis had a left-sided skew, and AA was bimodal (Figure 1).

## Discussion

This study summarizes the frequency of case reports of inflammatory skin diseases and describes their demographic distributions. Although some findings are in line with published studies, such as the decreasing prevalence of AD with age, or the congruent age distribution of AD and AA, which can co-occur in a large proportion of patients, other aspects were less representative, with the cases focusing more on male patients despite a generally female predominance in AD [5]. Additionally, there were over 11 times more cases reported about psoriasis than AA, despite prevalence estimates that are closer in magnitude. To the best of our knowledge, there is no clear reason why there would be such stark variations in gender demographics or disease representation. These results suggest that case reports may not be entirely reflective of the demographic makeup of different diseases. Study limitations include the use of automated review technologies that may lead to some missed case reports and that race and ethnicity were not available data points. Further research is needed to better understand how demographic representation is produced in inflammatory skin disease case reports.

## Abbreviations

AA: alopecia areata
AD: atopic dermatitis
PMC-Patients: PubMed Central Patients
